# Inter-Individual Responses to Experimental Muscle Pain: Baseline Physiological Parameters Do Not Determine Whether Muscle Sympathetic Nerve Activity Increases or Decreases During Pain

**DOI:** 10.3389/fnins.2015.00471

**Published:** 2015-12-17

**Authors:** Sophie Kobuch, Azharuddin Fazalbhoy, Rachael Brown, Vaughan G. Macefield

**Affiliations:** ^1^School of Medicine, Western Sydney UniversitySydney, NSW, Australia; ^2^Neuroscience Research AustraliaSydney, NSW, Australia; ^3^School of Health Sciences, RMIT UniversityBundoora, VIC, Australia

**Keywords:** blood pressure, HRV, MSNA, muscle pain, muscle sympathetic nerve activity

## Abstract

We have previously reported that there are inter-individual differences in the cardiovascular responses to experimental muscle pain, which are consistent over time: intramuscular infusion of hypertonic saline, causing pain lasting ~60 min, increases muscle sympathetic nerve activity (MSNA)—as well as blood pressure and heart rate—in certain subjects, but decrease it in others. Here, we tested the hypothesis that baseline physiological parameters (resting MSNA, heart rate, blood pressure, heart rate variability) determine the cardiovascular responses to long-lasting muscle pain. MSNA was recorded from the common peroneal nerve, together with heart rate and blood pressure, during a 45-min intramuscular infusion of hypertonic saline solution into the tibialis anterior of 50 awake human subjects (25 females and 25 males). Twenty-four subjects showed a sustained increase in mean amplitude of MSNA (160.9 ± 7.3%), while 26 showed a sustained decrease (55.1 ± 3.5%). Between the increasing and decreasing groups there were no differences in baseline MSNA (19.0 ± 1.5 vs. 18.9 ± 1.2 bursts/min), mean BP (88.1 ± 5.2 vs. 88.0 ± 3.8 mmHg), HR (74.7 ± 2.0 vs. 72.8 ± 1.8 beats/min) or heart rate variability (LF/HF 1.8 ± 0.2 vs. 2.2 ± 0.3). Furthermore, neither sex nor body mass index had any effect on whether MSNA increased or decreased during tonic muscle pain. We conclude that the measured baseline physiological parameters cannot account for the divergent sympathetic responses during tonic muscle pain.

## Introduction

Pain is important for survival by helping to avoid tissue damage, mobilizing all relevant homeostatic systems for a fight-and-flight response or, alternatively, promoting conservation of energy, and thus promoting healing (Craig, 2002). It is well known that pain originating in deep structures may evoke very different behavioral and cardiovascular responses than pain originating in superficial structures. Indeed, Lewis (1942) observed that pain originating in skin evokes “a rise of pulse rate” and a “sense of invigoration” whereas pain originating in deep structures evokes quiescence, a “slowing of the pulse” and “falling of the blood pressure” (Lewis, 1942). Subsequent studies confirmed Lewis' findings that muscle pain was associated with a fall in blood pressure and bradycardia in awake human subjects (Feinstein et al., 1954). However, since these early observations by Lewis and Feinstein, very few studies have examined the effects of pain on the cardiovascular system in awake human subjects.

We have been using subcutaneous or intramuscular injection of hypertonic saline—a specific stimulus for nociceptors (Graven-Nielsen and Mense, 2001)—to study the effects of acute pain on the cardiovascular system in awake human subjects. We showed that a bolus (0.5 ml) injection of hypertonic saline into the tibialis anterior muscle caused a *sustained* increase in muscle sympathetic nerve activity (MSNA), and a modest increase in blood pressure and heart rate (Burton et al., 2009a), while there was only a *transient* increase in skin sympathetic nerve activity (SSNA)—the latter being consistent with an arousal rather than reflex response (Burton et al., 2009b). More recently, we used intramuscular infusion to produce a sustained, steady-state, level of pain lasting for approximately 1 h (Fazalbhoy et al., 2012, 2014). We showed that about half of the subjects showed a sustained increase in MSNA, blood pressure, and heart rate during tonic muscle pain, while the other half showed sustained decreases (Fazalbhoy et al., 2012, 2014).

These data call into question the idea that noxious stimuli produce invariant responses and raise the prospect that these differential responses may be related to an individual's particular traits, which may be reproducible over time. That is, in some individuals muscle pain *always* evokes increases in MSNA, blood pressure and heart rate, whereas in others it consistently evokes decreases. Indeed, we recently showed that subjects who generate increases in MSNA, blood pressure and heart rate during one session also show increases in a second session; the same is true for those who show parallel decreases in MSNA, blood pressure and heart rate (Fazalbhoy et al., 2014). Moreover, we showed that there were no differences in resting MSNA, blood pressure or heart rate between the two recording sessions (Fazalbhoy et al., 2014), but we do not know whether differences in these baseline physiological parameters *across* individuals determines whether MSNA increases or decreases during tonic muscle pain. Indeed, in our first study we showed that resting levels of MSNA were higher in the group that showed an increase in MSNA than in the group that showed a decrease, but these differences failed to reach statistical significance—presumably because of the low subject numbers (*n* = 12) Against this background, the aim of the current study was to determine whether baseline physiological parameters—including resting MSNA, blood pressure and heart rate—could account for the divergent MSNA responses to tonic muscle pain. Our earlier studies (Fazalbhoy et al., 2012, 2014) were based on small subject numbers and were not sex-balanced. Here, we have studied a larger sample (*n* = 50), comprising 25 males and 25 females.

## Methods

Experiments were performed on 25 female and 25 male healthy subjects, aged 18–39 years. Data from 35 new participants were pooled with those from 15 participants reported previously (Fazalbhoy et al., 2014). All subjects provided informed written consent to the experimental procedures, which were conducted under the approval of the Human Research Ethics Committee of the University of Western Sydney and satisfied the requirements of the Helsinki Declaration. No subject had a history of cardiovascular disease or former chronic musculoskeletal pain. Prior to commencement height, weight, body mass index (BMI), and total muscle mass were measured for each subject using a body-composition analyser (SA165A, Tanita, Japan).

### Experimental procedures

The subjects were seated in a comfortable reclined position with the legs supported in an extended position. The room was kept quiet and at a constant temperature of 22°C. The course of the common peroneal nerve was identified via external stimulation (2–10 mA) using a 1 mm surface probe which delivered 0.2 ms pulses at 1 Hz from an isolated stimulator (Stimulus Isolator; ADInstruments, Sydney, Australia). Spontaneous bursts of muscle sympathetic nerve activity (MSNA) were recorded from muscle fascicles of the common peroneal nerve supplying the ankle or toe extensor or foot everter muscles via tungsten microelectrodes (FHC, Bowdoin, ME, USA) inserted percutaneously at the level of the fibular head. Multi-unit neural activity was amplified (gain 20 000, bandpass 0.3–5.0 kHz) using an isolated amplifier (NeuroAmp EX, ADInstruments, Sydney, Australia) and stored on computer (10-kHz sampling) using a computer-based data acquisition and analysis system (PowerLab 16SP hardware and LabChart 7 software; ADInstruments, Sydney, Australia). ECG (0.3–1.0 kHz) was recorded with Ag–AgCl surface electrodes on the chest and sampled at 2 kHz. Blood pressure was recorded continuously using finger pulse plethysmography (Finometer Pro, Finapres Medical Systems, The Netherlands) and sampled at 400 Hz. Respiration (DC-100 Hz) was recorded using a strain-gauge transducer (Pneumotrace, UFI, Morro Bay CA, USA) wrapped around the chest.

### Noxious stimulation

A 7% hypertonic saline solution was prepared by diluting sterile, 20% hypertonic saline with sterile water. Two syringes of 10 ml each were filled with the 7% hypertonic saline, placed in an infusion pump (Harvard Instruments, USA), and connected to a three-way tap via a 75 cm extension tubing primed with hypertonic saline. A 23 gauge butterfly needle was then attached to the three-way tap via a cannula, primed, and inserted 1.5 cm deep into the belly of the ipsilateral tibialis anterior muscle, about 5 cm lateral and 10 cm inferior to the tibial tuberosity. The cannula was inserted as soon as a stable recording of spontaneous MSNA was achieved. Prior to infusion of the saline solution, a 5 min baseline recording of MSNA, blood pressure, respiration, and heart rate was obtained. Infusion of the 7% hypertonic saline solution was started at a time unknown to the subject, and was maintained for 45 min; as described previously (Fazalbhoy et al., 2012, 2014), the pain lasted for ~60 min. The initial rate of infusion was set at 0.25 ml/min and was constantly adjusted to maintain a pain level of 5–6/10 on a Numerical Rating Scale (NRS). Subjects were asked to rate their pain continuously by sliding a linear potentiometer (Response Meter, ADInstruments, Sydney, Australia) that was calibrated to the NRS, with a rating of “0” meaning “no pain/discomfort” at all, and a rating of “10” being equivalent to the “worst pain the subject ever had experienced.” When the pain level dropped below 4/10 or rose above 6/10, the infusion rate was changed by 0.02 ml/min accordingly. After the infusion was completed, the recording was continued until the pain stopped. At the conclusion of the experiment, each subject completed a McGill Pain Questionnaire, in which subjects described the quality of the pain using a standard set of descriptors.

### Analysis

LabChart 7 Pro software (ADInstruments, Sydney, Australia) was used to record the following parameters: muscle sympathetic nerve activity (burst amplitude and frequency), heart rate, blood pressure, respiration, pulse pressure, heart rate variability (HRV), and pain ratings. Individual bursts of MSNA were displayed as a mean-voltage neurogram, computed as the root-mean-square (RMS) processed signal with a moving time average window of 200 ms. This signal was then analyzed using the “Peak Analysis” module of the LabChart 7 Pro software to calculate the amplitude of each burst. The absolute values were averaged into 5-min blocks and reported as percentages from the “baseline” values. An average of all blocks was taken to determine the direction of the response. Subjects with overall average MSNA amplitude 10% lower than baseline were arbitrarily assigned to the decreasing group; averages 10% higher than baseline were considered as increasing. Baseline MSNA amplitude was compared to the 5-min block with the mean value calculated over the entire infusion period, and to the highest average for the increasing group and to the lowest average value for the decreasing group. Changes in mean heart rate and mean blood pressure were also measured in 5 min epochs, normalized to the baseline value prior to the infusion of hypertonic saline. HRV was assessed over a 5-min steady state period before the infusion, and then again over 5 min when the subject experienced a steady-state level of pain during the infusion. The parameters of HRV that were analyzed included the low frequency (LF) and high frequency (HF) power, as well as the Root Mean Square Successive Difference of cardiac intervals (RMSSD). Statistical analysis—non-paired two-tailed *t*-tests for normally distributed data and Mann-Whitney tests for non-normally distributed data—was performed using Prism version 6 for Mac OS X (GraphPad software, San Diego, California, USA). All values are expressed as means and standard error. Probability levels of *p* < 0.05 were deemed significant.

## Results

### Subjective experience of tonic muscle pain

In all subjects intramuscular infusion of hypertonic saline induced a steady state level of muscle pain in the tibialis anterior muscle. The level of pain was kept constant, typically around 5 out of 10—throughout the period of infusion by adjusting the rate of infusion according to the subject's tracking of the pain level. The mean pain rating was 4.9 ± 0.1. Using the McGill Pain Questionnaire, 36 of the 50 subjects (72%) described the pain as “aching,” 48% described it as “heavy” and 48% as “dull.” After these, “throbbing,” “cramping,” “hurting,” “discomforting,” and “continuous” were the most frequent descriptions used.

### Muscle sympathetic nerve activity during tonic muscle pain

**Figure 1:**
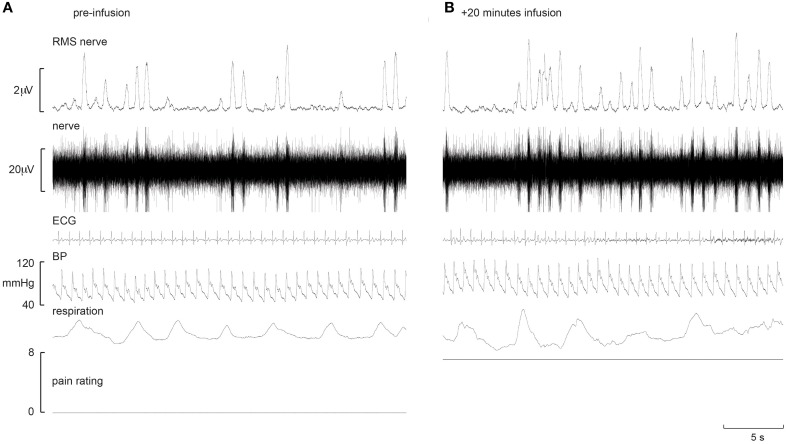
**Experimental records from one subject in whom MSNA increased during intramuscular infusion of hypertonic saline**. Baseline is shown in the left panel **(A)**, while the right panel **(B)** shows a sample at which MSNA was at its maximum.

**Figure 2:**
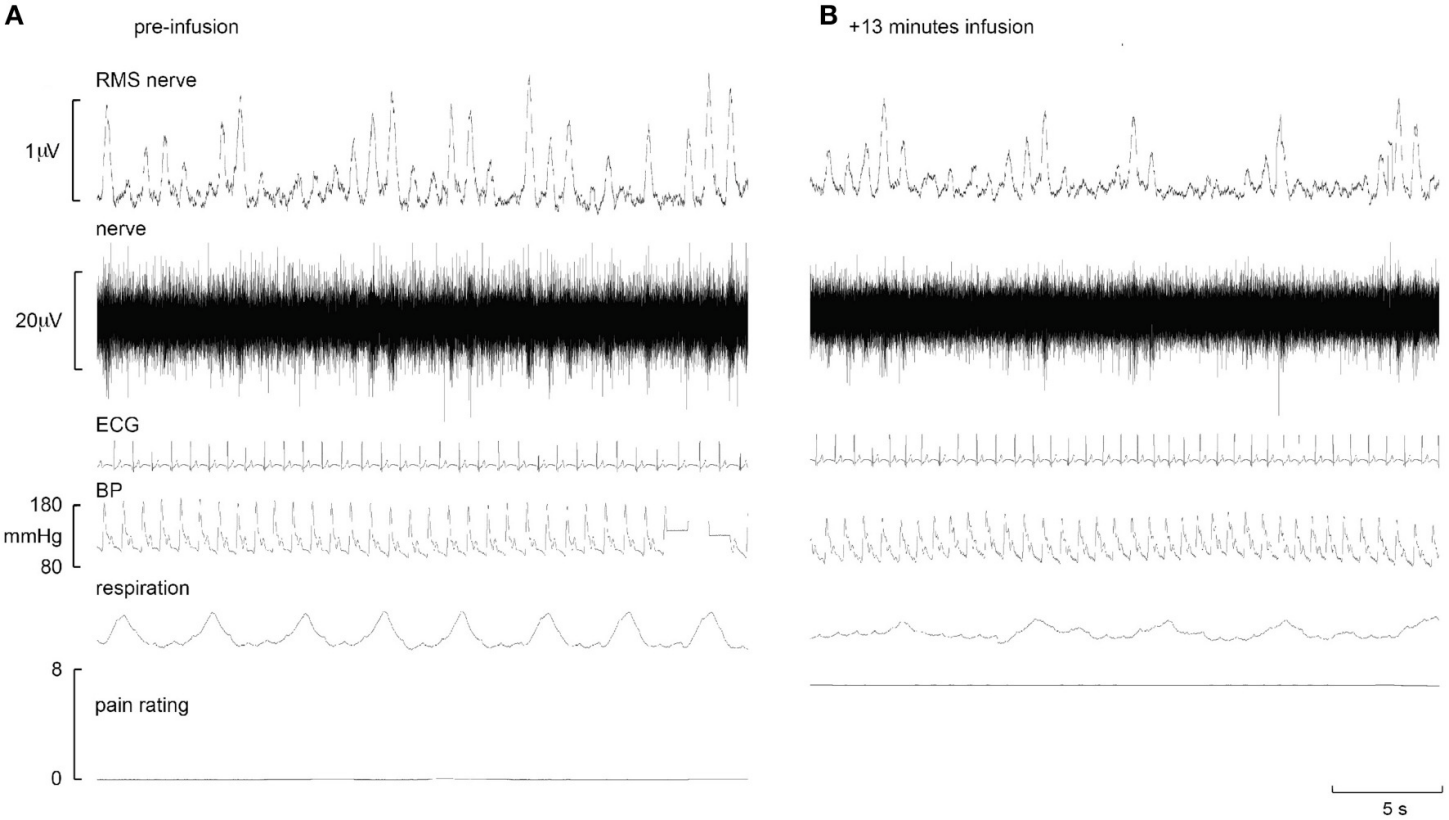
**Experimental records from one subject in whom MSNA decreased during intramuscular infusion of hypertonic saline**. Baseline is shown in the left panel **(A)**, while the right panel **(B)** shows a sample at which MSNA was at its maximum.

Experimental records from two subjects are shown in Figures 1, 2. Muscle sympathetic nerve activity (MSNA) increased during tonic pain in the subject depicted in Figure 1; it is apparent that blood pressure also increased. Conversely, the subject illustrated in Figure 2 exhibited a sustained decrease in MSNA and blood pressure during the infusion.

As expected, when all subjects were analyzed according to their pattern of MSNA response to muscle pain two distinct groups of responses emerged: 24 subjects (48%) showed a significant increase in burst amplitude over the entire infusion period (132.6 ± 6.1% *p* < 0.0001, *t*-test), while 26 subjects (52%) showed a significant decrease (72.6 ± 3.0%, *p* < 0.0001, *t*-test), relative to baseline. The peak changes in the increasing and decreasing groups, measured over 5 min, were 160.9 ± 7.3% and 55.1 ± 3.5% respectively; these were significantly different from baseline (*p* < 0.0001, *t*-test). The time at which the peak increase in MSNA occurred (29 ± 3 min) in the increasing group, and the time at which the peak fall occurred (32 ± 3 min) in the decreasing group, were not significantly different (*p* = 0.5077, Mann-Whitney test). There was no significant difference in the mean pain rating in the increasing and decreasing groups (4.7 ± 0.2 vs. 5.1 ± 0.2, respectively; *p* = 0.18, *t*-test).

### Blood pressure and heart rate during tonic muscle pain

**Figure 3:**
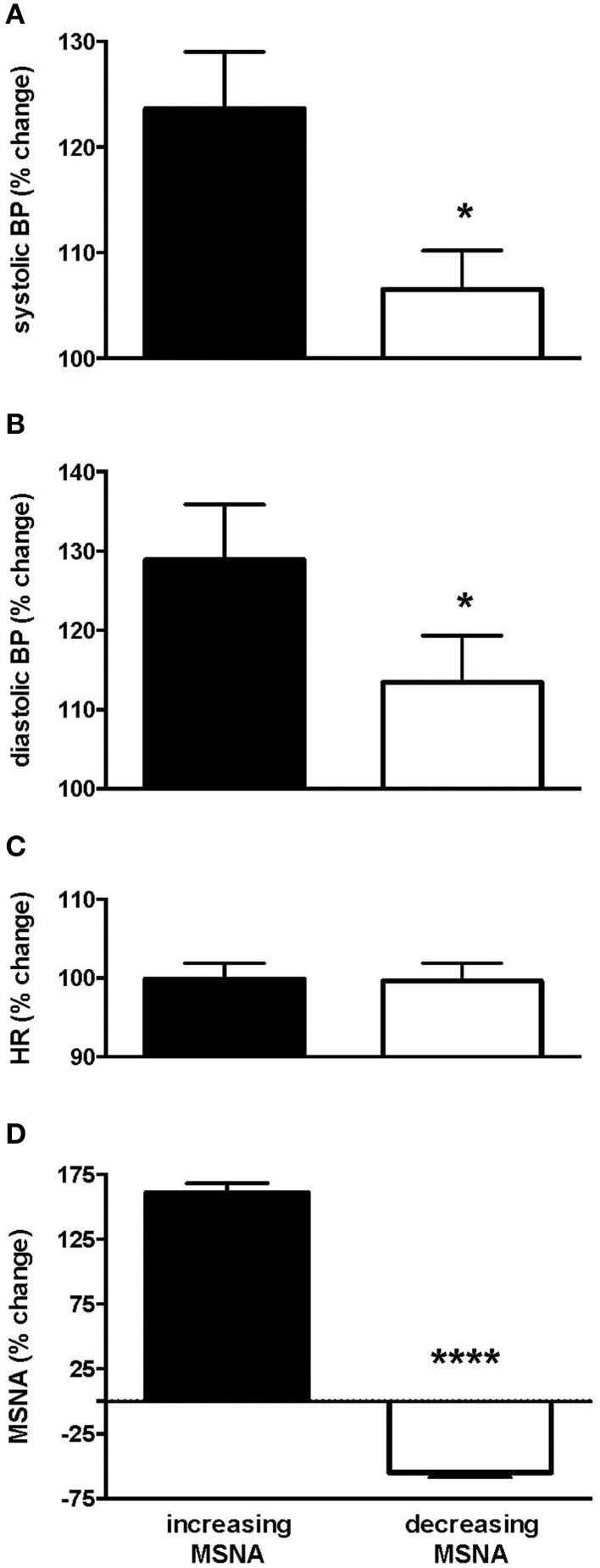
**Changes in systolic (A) and diastolic (B) pressure, heart rate (C) and muscle sympathetic nerve activity (MSNA; D) in the group of subjects in whom MSNA amplitude increased during tonic muscle pain and the group in whom MSNA amplitude decreased, measured from the peak changes**. Systolic and diastolic pressures were significantly higher in the increasing group as depicted by the asterisk. Results are compared to baseline levels (i.e., 100%).

Interestingly, those subjects who showed an increase in MSNA showed a significantly larger increase in blood pressure than those in whom MSNA decreased. Systolic pressure increased from 132.0 ± 5.5 (baseline) to 159.9 ± 5.8 mmHg (steady level of pain) in the increasing group but from only 133.0 ± 4.7 to 142.7 ± 5.3 in the decreasing group. Diastolic pressure increased from 70.2 ± 5.2 (baseline) to 86.6 ± 4.5 mmHg (steady level of pain) and from 75.1 ± 4.2 to 76.7 ± 4.3 in the increasing and decreasing groups, respectively. Relative changes in blood pressure, heart rate and MSNA in the two groups are presented in Figure 3. In the increasing group, data from two subjects were excluded from the calculated mean of all parameters as they showed much larger increases in amplitude of MSNA (396 and 520%), as defined by running an Outliers Test (Prism, GraphPad software), which would have skewed the results.

### Resting levels of MSNA and BP

When comparing the increasing and decreasing groups, there were no differences in baseline MSNA (19.0 ± 1.5 vs. 18.9 ± 1.2 bursts/min; *p* = 0.99, *t*-test) that could account for these divergent responses. Moreover, as shown in Table 1, there were no differences in resting blood pressure parameters, heart rate or heart rate variability, and no effect of body mass index or total muscle mass.

**Table 1:** **Baseline data for the group showing an increase in MSNA (***n*** = 24) during tonic muscle pain and the group showing a decrease (***n*** = 26)**.

	**Increasing MSNA**	**Decreasing MSNA**	***P*-value**
Number of subjects	11female±13male	14female±12male	0.78
Age (years)	22.1±1.3	22.4±0.9	0.34
Height (cm)	168.7±1.6	170.1±2.1	0.60
Weight (kg)	65.7±2.6	68.0±2.9	0.56
BMI (kg/m^2^)	23.1±1.0	23.4±0.8	0.65
Muscle mass (kg)	49.5±2.2	48.7±2.3	0.80
Pain rating (/10)	4.7±0.2	5.1±0.2	0.18
MSNA (bursts/min)	19.0±1.5	18.9±1.2	0.99
SAP (mmHg)	132.0±5.5	133.0±4.7	0.52
DAP (mmHg)	70.2±5.2	75.1±4.2	0.32
MAP (mmHg)	88.1±5.2	88.0±3.8	0.78
HR (beats/min)	74.7±2.0	72.8±1.8	0.44
LF HRV (nu)	56.9±3.8	59.4±4.0	0.80
HF HRV (nu)	38.4±3.4	35.6±3.7	0.58
LF/HF HRV	1.8±0.2	2.2±0.3	0.38
RMSSD HRV (ms)	40.5±4.1	40.8±4.0	0.99

*There were no statistically significant differences in age, height, body-mass index (BMI), total muscle mass between the two groups, or in baseline levels of MSNA, systolic arterial pressure (SAP), diastolic arterial pressure (DAP), mean arterial pressure (MAP), heart rate (HR) and specific components of heart rate variability (HRV; see Methods). Non-paired tests for all parameters except “number of subjects” (Fisher's exact test); nu, normalized units*.

### Sex differences

**Figure 4:**
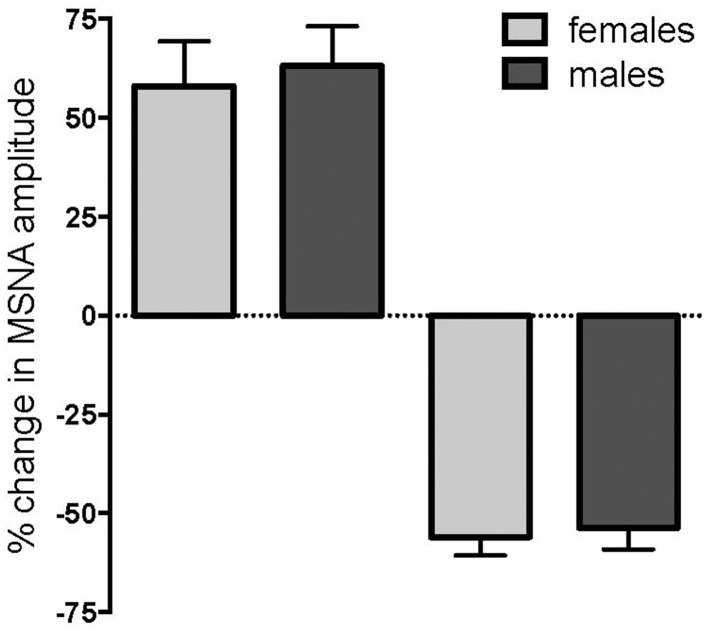
**Changes in muscle sympathetic nerve activity (MSNA) in females and males during tonic muscle pain**. There was no difference between females (light gray) and males (dark gray) in the propensity to show an increase or decrease in MSNA, and no differences in the magnitudes of the peak changes during muscle pain.

Of the 24 subjects in whom MSNA increased, 11 were female and 13 were male, while there were 14 females and 12 males in whom MSNA decreased. These data indicate that there was no difference in the *propensity* of males or females to exhibit an increase or decrease in MSNA during long-lasting muscle pain (*p* = 0.78, Fisher's Exact test). Moreover, the data illustrated in Figure 4 show that there were no differences in the peak *magnitude* of change in MSNA between females and males in either the increasing group (158.0 ± 11.3% vs. 163.2 ± 10.0%; *p* = 0.40, Mann-Whitney) or the decreasing group (44.1 ± 4.7% vs. 46.4 ± 5.4%; *p* = 0.77, Mann-Whitney). There was no significant difference in the mean pain rating between females and males (4.9 ± 0.2 vs. 4.9 ± 0.2, respectively; *p* = 0.87, *t*-test).

There were no statistically significant differences in resting MSNA between the female and male subjects (18.8 ± 1.5 vs. 20.2 ± 1.5 bursts/min; *p* = 0.19, unpaired Mann-Whitney test), and no significant differences in any of the other baseline cardiovascular parameters (Table 2). The only statistically significant differences between males and females were in BMI and muscle mass, both of which were significantly higher in the males (*p* = 0.05 and *p* < 0.0001, respectively), and age (*p* < 0.01)—on average, the females were one year older, though this is of no consequence because ages were not significantly different in the increasing and decreasing groups (cf Table 1).

**Table 2:** **Age, body-mass index (BMI), total muscle mass, MSNA frequency (normalized to baseline), systolic arterial pressure (SAP), diastolic arterial pressure (DAP), mean arterial pressure (MAP), heart rate (HR) and specific components of heart rate variability (HRV; see Methods) at baseline, divided by sex**.

	**Females**	**Males**	***P*-value**
Number of subjects	25	25	
Age (years)	22.8±0.8	21.8±1.5	<0.01
Height (cm)	164.0±1.1	175.9±1.7	<0.0001
Weight (kg)	61.5±2.7	72.6±2.2	<0.02
BMI (kg/m^2^)	22.9±1.0	23.4±0.5	0.05
Muscle mass (kg)	43.3±0.8	57.8±2.1	<0.0001
Pain rating (/10)	4.9±0.2	4.9±0.2	0.87
MSNA (bursts/min)	18.8±1.5	20.2±1.5	0.19
SAP (mmHg)	131.7±6.3	133.3±3.3	0.96
DAP (mmHg)	72.7±5.4	72.7±3.9	0.69
MAP (mmHg)	89.5±5.5	86.6±3.3	0.96
HR (beats/min)	75.0±1.9	72.3±1.9	0.32
LF HRV (nu)	61.7±3.5	57.4±3.2	0.21
HF HRV (nu)	33.8±3.2	37.2±3.0	0.16
LF/HF HRV	2.2±0.3	1.8±0.3	0.15
RMSSD HRV (ms)	39.0±3.9	41.9±4.5	0.64

*Non-paired tests for all parameters; nu, normalized units*.

## Discussion

This study extends the recent work conducted in our laboratory on the effects of experimental muscle pain on the sympathetic nervous system (Burton et al., 2008, 2009a,b; Fazalbhoy et al., 2012, 2014; Hall et al., 2012). In our first study of 12 subjects we found that tonic muscle pain, produced by intramuscular infusion of hypertonic saline for 45 min created divergent changes in muscle sympathetic outflow: one group (*n* = 7) showing an increase in MSNA and another (*n* = 5) showing a decrease (Fazalbhoy et al., 2012). In a second study of 15 subjects, we had reported that 11 subjects showed consistent increases (*n* = 5) or decreases (*n* = 6) in MSNA when assessed on two occasions at least 2 weeks apart (Fazalbhoy et al., 2014). Here we have confirmed the findings of divergent sympathetic responses to long-lasting muscle pain, but with a much larger sample size (*n* = 50): one group of people (*n* = 24) showed an increase in MSNA and another group (*n* = 26) showed a decrease.

### Baseline physiological parameters

The findings of the current study suggest that the cardiovascular responses to long-lasting muscle pain are not determined by our measured baseline physiological levels; both the direction of the response and the magnitude of change were independent of baseline MSNA, heart rate, blood pressure, heart rate variability, as well as age, sex, and BMI. This is consistent with studies showing comparable control and sensitivity of the sympathetic baroreflex in young men and young women (Tank et al., 2005; Studinger et al., 2009; Hart et al., 2011). Whether, these findings remain with increasing age is beyond the scope of this study. However, it would be interesting to know whether the pattern of response remains unchanged with age.

In this larger sample of subjects we found no correlation between MSNA and heart rate, unlike the parallel changes observed in the smaller data sets reported previously (Fazalbhoy et al., 2012, 2014). Because of the dual innervation of the heart, it may well be that the increase in sympathetic outflow to the vascular bed in muscle is matched by a parallel increase in cardiac sympathetic drive, which would increase heart rate, but that a competing parasympathetic influence via the vagus nerve counteracts this.

### MSNA and blood pressure

Although, there was no difference in the changes in heart rate and the change in MSNA between the two groups, in the group of subjects in whom MSNA increased during tonic muscle pain blood pressure was significantly higher than in the group in whom MSNA decreased. This suggests that the increase in MSNA was driving the increase in blood pressure, as an increase in blood pressure should, via the baroreflex, lead to a fall in MSNA. Indeed, the latter mechanism may explain why in some subjects MSNA fell despite an increase in blood pressure: in these cases, it would appear that the increase in blood pressure was causing a baroreflex-mediated reduction in MSNA, while in other instances a reduction in both blood pressure and MSNA could be the result of a nociceptor-driven withdrawal of MSNA. However, for those subjects in whom both MSNA and blood pressure increased during tonic muscle pain, we would like to suggest that nociceptor-driven increases in blood pressure could potentially be a risk factor for the development of clinically significant high blood pressure in the future, given that some individuals with chronic pain go on to develop hypertension. Indeed, patients with post-surgical chronic pain have nearly twice the prevalence of clinical hypertension than medical patients without pain (Bruehl et al., 2005). Accordingly, we could postulate that a person who consistently exhibited increases in MSNA, blood pressure, and heart rate during experimental muscle pain may—if he or she developed chronic pain from an injury in the future—go on to develop hypertension.

### Heart rate variability

Heart rate variability is widely reported to reflect the degree of sympathetic and parasympathetic control over the heart. The LF band is proposed to represent (primarily) sympathetic cardiac activation (Malliani et al., 1991), while the HF band is proposed to reflect vagal cardiac control (Bernston et al., 1997). Subsequently, the LF/HF ratio has been suggested as an index of the sympathovagal balance (Cohen et al., 2000; Martinez-Lavin, 2004; Staud, 2008; Reyes del Paso et al., 2011). The value of HRV in distinguishing between cardiac sympathetic and parasympathetic outflow is debatable (Goldstein et al., 2011). However, that there was no difference between any HRV parameters at either baseline or during tonic pain indicates that HRV is not related to whatever is responsible for the divergent sympathetic responses to muscle pain seen in this study.

### Limitations

The intramuscular infusion of hypertonic saline occurred at a time unknown to the subject, who was asked to continuously report the development of pain, as a rating out of 10, via the linear potentiometer provided. Infusion rates were titrated—by increasing or decreasing the rate of infusion in increments of 0.02 ml/min—to maintain a constant level of pain. Although we did not routinely record either the rate of infusion, or the total volume infused, in each subject, we never exceeded 20 ml (as noted in Methods we used two syringes of 10 ml each). Nevertheless, there were no differences in total muscle mass in the group in whom MSNA increased and the group in whom MSNA decreased and, given that the infusion caused a notable distension of the muscle belly in both groups, it is reasonable to assume that there any changes in plasma osmolality were limited to the muscle compartment and that comparable depolarization of small-diameter axons by the hypertonic saline occurred in the two groups. In other words, the noxious sensory input was the same in the two groups, as reflected in the fact that there were no significant differences in mean pain ratings between the two groups. The same was true when we separated the cohort into males and females: the only significant differences here being the higher BMI and lower total muscle mass in the females, both of which are expected. Of course, one could argue that the intramuscular infusion of hypertonic saline would have a greater effect in a smaller muscle (in the females), but in our experience we see no differences in mean pain ratings in small muscles (e.g., intrinsic muscles of the hand) and large muscles (e.g., flexor carpi radialis, deltoid, tibialis anterior), and pain ratings were the same in males and females.

### Implications

We have shown, in a large sample of subjects (*n* = 50), that the baseline physiological parameters measured here do not predict whether an individual exhibits an increase or decrease in MSNA during long-lasting muscle pain. Furthermore, sex appears to play no role in determining the direction of response to muscle pain. Unlike the short-lasting pain we had previously induced by bolus injections (Burton et al., 2009a,b), we believe the physiological responses to tonic pain will more closely replicate episodes during which chronic pain patients are suffering and coping with their pain. Persistent deep pain in experimental animals has been shown to provoke a passive coping response—i.e., conservation/withdrawal (Keay and Bandler, 2002). Of course, while tonic muscle pain lasting only 20 min has been used as a model for chronic musculoskeletal pain (Capra and Ro, 2004), we should stress that this only reflects continuous nociceptive pain and not the neuropathic pain typically associated with chronic pain. Nevertheless, this method of inducing pain offers the advantage of allowing a controlled investigation into how pain may modulate MSNA, blood pressure, and heart rate. Conversely—assuming everything else is equal—one would need to know the level of MSNA in a person prior to the development of chronic pain in order to interpret any changes in muscle sympathetic outflow. Microelectrode recordings of sympathetic nerve traffic in human subjects have found no differences in sympathetic outflow to a painful limb compared to the contralateral non-painful limb in patients with complex regional pain syndrome, suspected to be sympathetically maintained because of the marked cutaneous vasoconstriction (Casale and Elam, 1992). In order to understand the neurophysiological basis of the divergent sympathetic responses to experimental muscle pain, further investigations are needed, as the current results fail to demonstrate that baseline physiological parameters, BMI or sex, play a role in the cardiovascular responses to long-lasting muscle pain in humans.

### Conflict of interest statement

The authors declare that the research was conducted in the absence of any commercial or financial relationships that could be construed as a potential conflict of interest.
